# Climbing behavior by mice as an endpoint for preclinical assessment of drug effects in the absence and presence of pain

**DOI:** 10.3389/fpain.2023.1150236

**Published:** 2023-04-17

**Authors:** Edna J. Santos, Arianna N. Giddings, Farah A. Kandil, S. Stevens Negus

**Affiliations:** Department of Pharmacology and Toxicology, School of Medicine, Virginia Commonwealth University, Richmond, VA, United States

**Keywords:** pain, ICR mice, climbing, opioids, efficacy

## Abstract

This study evaluated climbing in mice as a tool to assess the expression and treatment of pain-related behavioral depression in male and female ICR mice. Mice were videotaped during 10-min sessions in a vertical plexiglass cylinder with wire mesh walls, and “Time Climbing” was scored by observers blind to treatments. Initial validation studies demonstrated that baseline climbing was stable across repeated days of testing and depressed by intraperitoneal injection of dilute lactic acid (IP acid) as an acute pain stimulus. Additionally, IP acid-induced depression of climbing was blocked by the positive-control non-steroidal anti-inflammatory drug (NSAID) ketoprofen but not by the negative control kappa opioid receptor agonist U69593. Subsequent studies examined effects of single-molecule opioids (fentanyl, buprenorphine, naltrexone) and of fixed-proportion fentanyl/naltrexone mixtures (10:1, 3.2:1, and 1:1) that vary in their efficacy at the mu opioid receptor (MOR). Opioids administered alone produced a dose- and efficacy-dependent decrease in climbing, and fentanyl/naltrexone-mixture data indicated that climbing in mice is especially sensitive to disruption by even low-efficacy MOR activation. Opioids administered as a pretreatment to IP acid failed to block IP acid-induced depression of climbing. Taken together, these findings support the utility of climbing in mice as an endpoint to evaluate candidate-analgesic effectiveness both to (a) produce undesirable behavioral disruption when the test drug is administered alone, and (b) produce a therapeutic blockade of pain-related behavioral depression. The failure of MOR agonists to block IP acid-induced depression of climbing likely reflects the high sensitivity of climbing to disruption by MOR agonists.

## Introduction

Clinically relevant pain is often associated with impaired function and behavioral depression, and a common goal of pain treatment is to alleviate these manifestations of pain and restore normal behavior (1, 2). Preclinical research using experimental pain models can focus on parallel endpoints of “pain-depressed behavior,” which can be defined as behaviors that decrease in rate, frequency, or intensity after delivery of a noxious stimulus (3, 4). There are two main advantages to studying pain-depressed behaviors as a category of endpoints for research on expression, mechanisms, and treatment of pain. First, preclinical-to-clinical translational research in any domain is optimized when preclinical studies measure endpoints homologous to clinically relevant human endpoints (5, 6), and as noted above, preclinical endpoints of pain-depressed behavior are homologous to clinical endpoints of pain-related functional impairment and behavioral depression (7). Second, pain-depressed behaviors are not susceptible to false positive effects observed with drugs that cause motor impairment because effective analgesics will increase the expression of the pain-depressed behaviors, whereas drugs that produce motor impairment only exacerbate pain-related behavioral depression (4, 8).

In an effort to develop valid and efficient assays of pain-depressed behavior, experimental models of acute and chronic pain have been evaluated for their effectiveness in rodents to decrease a range of different behaviors, including horizontal locomotion (9, 10), wheel running (7), and nesting (11, 12) by mice. The present study sought to evaluate pain-related depression of another behavior: climbing. Climbing is an ethologically important component of locomotor behavior in rodents (13, 14), and it consists of vertical locomotion required to navigate vertically oriented surfaces in the wild. However, climbing is rarely examined in laboratory environments, where home cages and behavioral testing chambers are usually shallow and have smooth walls that cannot be scaled. Other types of test environments with taller profiles and scalable vertical surfaces have occasionally been used to assess climbing in mice (15, 16), but these types of environments have not yet been used to assess the effects of experimental pain models in the absence or presence of known or candidate analgesics.

Accordingly, the goal of this study was to use a vertically oriented cylinder with wire-mesh walls as a test environment to assess the expression and treatment of pain-related depression of climbing in mice. Initial validation of the procedure proceeded in four steps. First, we evaluated the expression and stability of climbing during repeated, within-subject testing to assess suitability of climbing for a within-subjects experimental design. Second, we determined the effectiveness of intraperitoneal injection of dilute lactic acid (IP acid) as an acute noxious stimulus to decrease climbing. IP acid injection models tissue acidosis associated with many types of pain states (17), and we have shown previously that it produces a concentration-dependent depression of a wide range of different behaviors in mice and rats (8, 18, 19). Third, we evaluated the effects of the positive-control non-steroidal anti-inflammatory drug (NSAID) ketoprofen to block IP acid-depressed climbing. Ketoprofen is a clinically effective analgesic, and we have previously shown that it blocks IP acid-induced depression of a range of different behaviors in both mice and rats (9, 12, 18, 20). Finally, we evaluated the effects of the centrally acting kappa opioid receptor (KOR) agonist U69593 as a negative control. Centrally acting KOR agonists represent one class of candidate analgesics that has produced analgesia-like effects in conventional preclinical procedures but that has failed to produce reliable and safe analgesia in humans [e.g., (21)] and similarly fails to alleviate pain-related behavioral depression preclinically (18, 22–24).

Following the initial validation process, we investigated the role of mu-opioid receptor (MOR) ligand efficacy as a determinant of MOR agonist effectiveness to block IP acid-induced depression of climbing. High-efficacy MOR agonists like fentanyl and morphine are clinically effective analgesics, but their use is limited by side effects such as respiratory depression, impaired motor function, inhibition of gastrointestinal transit, tolerance, dependence, and abuse liability (25–27). Lower efficacy MOR agonists like buprenorphine retain clinically effective analgesic effects, but they produce fewer and weaker side effects and are therefore safer (28–30), but are rarely used (31). As a result, the development of novel, selective, low-efficacy MOR agonists may represent a promising path for analgesic drug development (32–34). MOR efficacy may be especially relevant for opioid effects in assays of pain-depressed behavior, where opioids can produce competing effects that include both analgesia (which alleviates pain-related behavioral depression and increases rates of the target behavior) and motor impairment (which can reduce rates of the target behavior and obscure analgesic restoration of pain-depressed behavior) (19, 34, 35). Accordingly, we manipulated MOR efficacy by testing both (a) a set of single-molecule opioids with decreasing MOR efficacy (fentanyl > buprenorphine > naltrexone) (34, 36, 37), and (b) a series of fixed-proportion fentanyl/naltrexone mixtures that vary in net MOR efficacy as described previously (36, 38–40). We hypothesized that low-efficacy single-molecule opioids or fentanyl/naltrexone mixtures would have sufficient efficacy to alleviate pain-related depression of climbing without affecting motor behavior.

## Methods and materials

### Subjects

Subjects were male and female ICR mice (Envigo, Frederick, MD) that were 6–8 weeks old upon arrival to the laboratory. ICR mice were used in this study because it is an outbred strain of mice and outbred strain of mice have been recommended as being advantageous in pain studies (41). Males weighed 27–50 g and females weighed 23–38 g throughout the study. Mice were generally housed in same-sex, littermate groups of three mice per cage with corncob bedding (Envigo), a “nestlet” composed of pressed cotton (Ancare, Bellmore, NY), a cardboard tube for enrichment, and *ad libitum* access to food (Teklad LM-485 Mouse/Rat Diet; Envigo). In some cases, males were split into smaller groups or isolated to minimize fighting. Cages were mounted in a RAIR HD Ventilated Rack (Laboratory Products, Seaford, DE) in a temperature-controlled room with a 12-hour light/dark cycle (lights on from 6:00 AM to 6:00 PM) in a facility approved by the American Association for Accreditation of Laboratory Animal Care. All experiments were performed during the light phase of the daily light/dark cycle beginning 1 week after arrival at the laboratory. Animal-use protocols were approved by the Virginia Commonwealth University Institutional Animal Care and Use Committee and complied with the National Research Council Guide for the Care and Use of Laboratory Animals.

### Apparatus and climbing assessment

**Figure 1:**
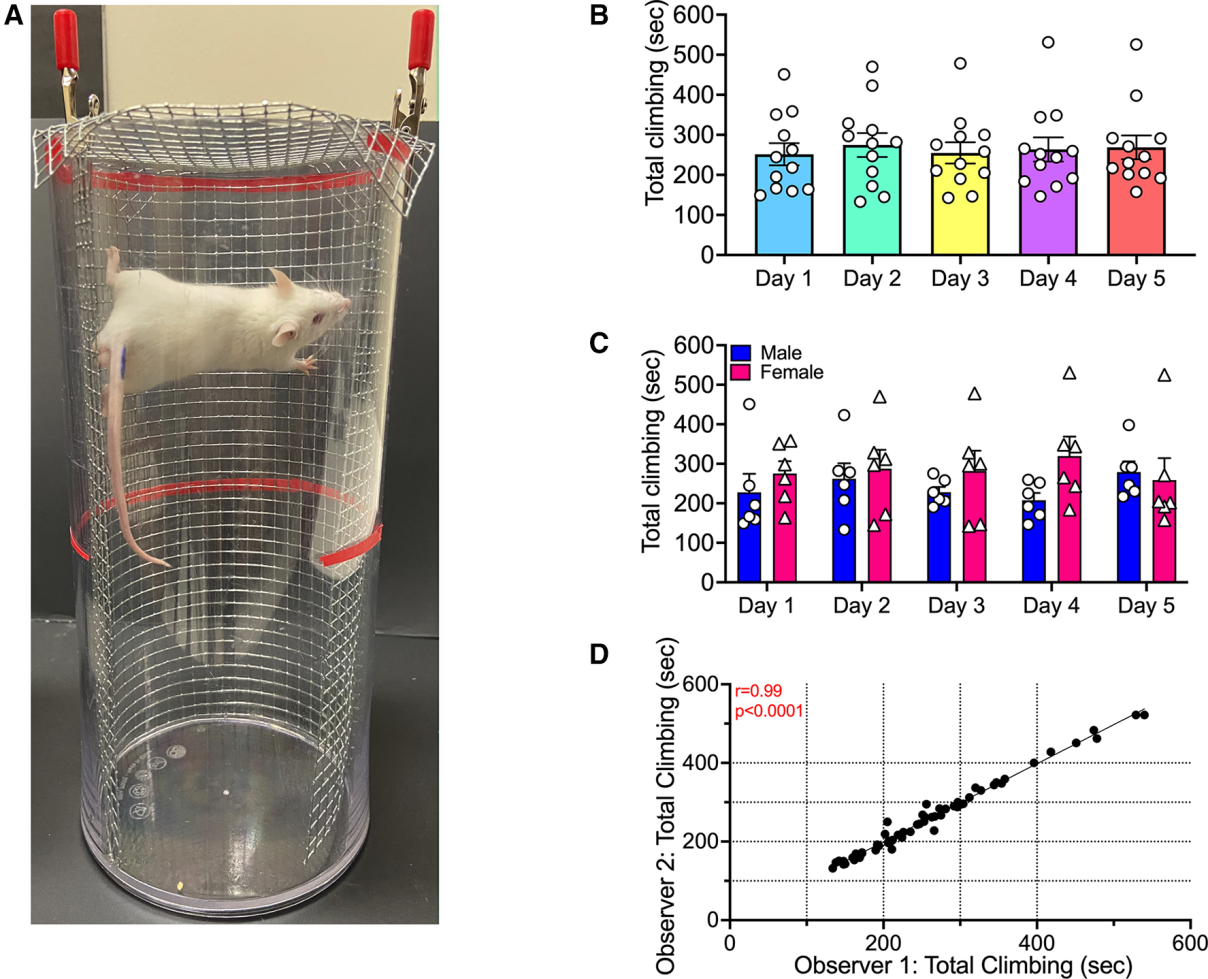
Climbing by mice during repeated testing. (**A**) The climbing apparatus. (**B**) Abscissa: Test day. Ordinate: Time climbing in sec. Each bar shows mean ± SEM from 12 mice (6 male, 6 female), and points show data for individual mice. (**C**) Same data as in panel B segregated by sex. (**D**) Inter-rater reliability of climbing times assigned by two different observers for all mice across all test days.

To assess climbing, mice were transported to an experimental room separate from the housing room and placed individually into clear plastic cylinders (11.25 cm diameter ×25.5 cm tall; see Figure 1) for 10-min behavioral sessions. Each cylinder was lined from bottom to top with 0.5 cm^2^ aluminum wire mesh around 75% of the inner perimeter (26.03 cm width ×24.13 cm height; no mesh in front to permit unobscured video recording). Additionally, the top of the cylinder was covered by a lid made from the same wire mesh. During each session, three mice were tested at once in separate cylinders. Cardboard barriers between the cylinders prevented visual contact between mice during testing, and behavior was recorded with a video camera (Amazon, Inc GordVE Video Camera Camcorder HD 1080P) or an iPad (Apple Inc, 2011) with the experimenter absent from the room. The main dependent variable was the amount of time mice spent climbing during each 10-min behavioral session. “Climbing” was defined as any time that a mouse had at least one paw in contact with the mesh wall or lid and all paws off the floor. Time climbing was scored by at least one of two trained observers blind to experimental treatments. A subset of videos was scored by both observers at the beginning of the study and periodically during the study to monitor inter-rater reliability.

### Experimental design and procedure

Studies proceeded in two phases to (1) validate the procedure, and (2) test the effects of mu opioid receptor (MOR)-ligand treatments designed to vary the efficacy of MOR activation. Each experiment was conducted using a within-subjects repeated-measures design. Treatments within each group were randomized across subjects using a Latin-square design, and tests were separated by three to four days to permit drug washout between tests.

Initial validation studies proceeded in four steps. Step 1 evaluated the stability of climbing during repeated testing. Mice in this group received no injections and were tested a total of five times at intervals of three to four days to mimic the testing intervals planned for subsequent treatment studies. Step 2 evaluated pain-related depression of climbing produced by intraperitoneal injection of dilute lactic acid (IP acid) as an acute visceral noxious stimulus. Mice in this group were tested with IP water or a range of IP acid concentrations (0.18%–0.56%) administered 10 min before each behavioral session. Step 3 evaluated effects of the clinically effective positive-control analgesic and non-steroidal anti-inflammatory drug ketoprofen (10 mg/kg) administered subcutaneously (SC) as a pretreatment to 0.32% IP acid. Mice in this group received four treatments: SC ketoprofen + IP acid, SC ketoprofen + IP water, SC saline + IP acid, or SC saline + IP water. SC ketoprofen or its vehicle was administered 30 min before the session, and IP acid or water was administered 10 min before the session. Step 4 evaluated effects of the negative-control kappa opioid receptor (KOR) agonist U69593 administered SC alone or as a pretreatment to 0.32% IP acid. One group of mice was used to evaluate effects of U69593 administered alone (vehicle and 0.1–1.0 mg/kg), and a second group of mice received U69593 (vehicle and 0.1–1.0 mg/kg SC) administered as a pretreatment before 0.32% IP acid. U69593 or its vehicle was administered 20 min before the session, and IP acid was administered 10 min before the session.

Studies to evaluate the effects of MOR activation proceeded in two steps. In Step 1, effects were determined for SC administration of the high-efficacy MOR agonist fentanyl (0.0032–0.1 mg/kg), the intermediate-efficacy MOR agonist buprenorphine (0.01–0.32 mg/kg), and the MOR antagonist naltrexone (0.01–0.1 mg/kg) and their saline vehicles. Each MOR ligand was evaluated both alone in one group of mice and as a pretreatment to 0.32% IP acid in a second group of mice. Step 2 evaluated effects produced by a series of fixed-proportion fentanyl/naltrexone mixtures. We have reported previously that the proportion of fentanyl in fentanyl/naltrexone mixtures can be manipulated such that decreasing fentanyl proportions result in decreasing net efficacy of the mixture (36, 38–40). Here, we examined 10:1, 3.2:1, and 1:1 mixtures of fentanyl/naltrexone. As with the single-molecule MOR ligands, each fentanyl/naltrexone mixture was evaluated both alone in one group of mice and as a pretreatment to 0.32% IP acid in a second group of mice. For all fentanyl/naltrexone mixture studies, the fentanyl doses were 0.0032–0.1 mg/kg, and the naltrexone doses varied according to the designated proportion. For all MOR ligands and mixtures, the opioid or its vehicle was administered 20 min before the session, and IP acid was administered 10 min before the session.

Mice were randomly assigned to treatment groups, and testing progressed until 12 mice (6 male, 6 female) met inclusion criteria for a given treatment. The only exception was the 10:1 fentanyl/naltrexone + IP acid group, which has data from 11 mice (6 male, 5 female). There were two inclusion criteria. First, all treatment groups included a vehicle control, and mice were included only if they climbed for ≥60 s under these control conditions. Second, for groups to examine test drug effects as pretreatments to IP acid, mice were included only if drug vehicle + IP acid produced ≥20% decrease in climbing time relative to vehicle treatment. The number of mice assigned to each group but failing to meet the inclusion criteria is reported for each group in Table 1.

**Table 1:** Summary of vehicle control data and one-way ANOVA results for each group tested in the present study. The sex of excluded mice is indicated by M (males) or F (females). NA, not applicable.

			Exclusions		
Treatment	Vehicle condition	Mean climbing (sec ± SEM) after vehicle	Vehicle climbing <60 sec	IP acid depression of climbing <20%	One-way ANOVA
No treatment	none	251.5 ± 27.75 (day 1)	–	–	F (2.77, 30.42) = 0.21; *P* = 0.8748
Lactic acid (LA)	IP H_2_O	159.2 ± 28.58	1 Male	–	F (2.30, 25.31) = 12.50; < 0.0001
Ketoprofen ± LA	SC Sal + IP H_2_O	160.9 ± 15.21	3 Female	1 Male 2 Female	F (2.51, 27.55) = 15.38; *P* < 0.0001
U69593	SC Sal	346.1 ± 44.25	–	NA	F (2.85, 65.62) = 21.29; *P* < 0.0001
Fentanyl	SC Sal	246.9 ± 26.88	1 Female	NA	F (2.63, 28.98) = 26.27; *P* < 0.0001
Buprenorphine	SC Sal	256.6 ± 43.47	–	NA	F (2.06, 22.70) = 10.55; *P* = 0.0005
Naltrexone	SC Sal	278.3 ± 29.64	–	NA	F (2.16, 23.76) = 0.60; *P* = 0.5715
FENT/NTX 10:1	SC Sal	245.4 ± 37.85	–	NA	F (2.17, 23.87) = 28.79; *P* < 0.0001
FENT/NTX 3.2:1	SC Sal	270.9 ± 14.53	–	NA	F (2.79, 30.74) = 19.95; *P* < 0.0001
FENT/NTX 1:1	SC Sal	236.1 ± 53.01	1 Male 1 Female	NA	F (2.59, 28.52) = 1.16; *P* = 0.3388
U69593 + LA	SC Sal + IP H2O	257.0 ± 48.13	2 Male	1 Female	F (2.58, 28.33) = 1.13; *P* = 0.3479
Fentanyl + LA	SC Sal + IP H2O	170.0 ± 19.99	1 Male	–	F (2.08, 22.83) = 0.91; *P* = 0.4184
Buprenorphine + LA	SC Sal + IP H2O	166.8 ± 20.71	1 Female	–	F (1.08, 11.91) = 0.87; *P* = 0.3781
Naltrexone + LA	SC Sal + IP H2O	230.0 ± 38.27	2 Male 1 Female	–	F (1.61, 17.76) = 1.11; *P* = 0.3381
FENT/NTX 10:1 + LA	SC Sal + IP H2O	208.9 ± 34.93	1 Female	–	F (1.62, 16.17) = 1.19; *P* = 0.3199
FENT/NTX 3.2:1 + LA	SC Sal + IP H2O	172.8 ± 40.94	1 Male 3 Female	1 Female	F (2.14, 23.59) = 0.70; *P* = 0.5178
FENT/NTX 1:1 + LA	SC Sal + IP H2O	201.8 ± 23.78	–	1 Male 2 Female	F (1.96, 21.51) = 2.63; *P* = 0.0963

### Data analysis

Behavioral sessions were videotaped and scored by trained observers blinded to experimental treatments. Raw data as “Time Climbing” in sec are reported for the first experiment to examine stability of climbing across days in mice that received no other treatment. For all subsequent analyses with IP acid and test drugs, data in each mouse were transformed to a % of the mean vehicle control data for that mouse's group using the equation (time climbing after a given treatment in a given mouse ÷ mean time climbing after vehicle control for that mouse's group) ×100. Raw data (for the first experiment) and transformed data (for all drug ± IP acid experiments) were analyzed in a series of three steps as described by us previously for studies that include both females and males but are not intended *a priori* to detect sex differences (42). First, because sex was not the primary variable of interest, pooled data from both females and males were analyzed by repeated-measures one-way ANOVA with time or dose as the single variable, and a significant ANOVA was followed by a Dunnett's or Tukey's *post hoc* test. Second, data were segregated by sex and analyzed by two-way ANOVA with sex as a between-subjects factor and time or dose as a within-subjects factor. A significant main effect of sex or sex x dose interaction was followed by a Holm-Sidak post-hoc test. These first two steps of data analysis were performed using GraphPad Prism 9.0 (La Jolla, CA). Lastly, the two-way ANOVA results were submitted to power analyses to calculate the Cohen's f effect size, achieved power (1–*β*), and the total number of animals predicted as necessary to achieve power ≥0.8 using the free statistical analysis program G*Power (43).

In addition to these within-group analyses, three types of between-group analyses were conducted. First, raw vehicle control data were compared by one-way ANOVA across groups receiving the three different types of vehicle control treatment: SC saline alone, IP water alone, or SC saline + IP water. A significant ANOVA was followed by a Holm-Sidak *post hoc* test to compare each group to all other groups. Second, raw vehicle control data were also compared by one-way ANOVA across individual groups for which the vehicle control was either SC saline alone or IP water administered alone or in conjunction with SC saline. A significant ANOVA was again followed by a Holm-Sidak *post hoc* test. Lastly, data from experiments with fentanyl/naltrexone mixtures administered alone were used to determine the efficacy requirement for opioid effects on climbing as we have described previously (36, 38–40). Briefly, data from each mixture were transformed to a percent of the maximum effect produced by fentanyl alone using the equation [(vehicle - mixture) ÷ (vehicle – fentanyl)] ×100, where “vehicle” equals the mean vehicle control data in a group, “mixture” equals the time climbing in a given mouse after a given dose of a mixture, and “fentanyl” equals the mean maximum effect of fentanyl alone in the fentanyl treatment group. The maximum effect of each mixture was then plotted as a function of the proportion of fentanyl in the mixture, and linear regression was used to determine the EP50 value (95% confidence limits), with EP50 defined as the “effective proportion” of fentanyl to naltrexone required to produce 50% of the maximum fentanyl-alone effect. The EP50 serves as a metric of the efficacy requirement for a given effect, and the EP50 for fentanyl/naltrexone-mixture effects in this study was compared to EP50 values determined in previous studies for fentanyl/naltrexone-mixture effects on other previously reported *in vivo* and *in vitro* endpoints. EP50 values were considered to be significantly different if 95% confidence limits did not overlap.

Two observers were trained to score all videos and most videos were scored by only one of these two observers; however, a subset of videos at the beginning of the study and periodically during the study were scored by both observers. Inter-rater reliability was assessed by determining the Pearson's r and *P*-value for the correlation in observer scores.

### Drugs

Fentanyl HCl, naltrexone HCl, buprenorphine HCl, and U69593 were provided by the National Institute on Drug Abuse Drug Supply Program and were dissolved in sterile saline. Ketoprofen (100 mg/ml; Ford Dodge, IA) was diluted in sterile saline. All drugs were administered subcutaneously (SC) in volumes of 10 ml/kg. Lactic acid was purchased from Sigma-Aldrich (St. Louis, MO), diluted in sterile water, and administered intraperitoneally (IP) in a volume of 10 ml/kg.

## Results

Figure 1 shows that climbing in the absence of any treatment was stable with repeated testing, and this figure also illustrates the statistical analysis pipeline for all subsequent experiments. First, data from both sexes were pooled and analyzed by repeated-measures one-way ANOVA (Figure 1B). This analysis indicated no effect of test day [F (2.766, 30.42) = 0.2100; *P* = 0.8748]. Second, data were segregated by sex and analyzed by two-way ANOVA (Figure 1C). This analysis indicated no main effect of either Day [F (2.330, 23.30) = 0.2168; *P* = 0.8378] or Sex [F (1, 10) = 0.9937; *P* = 0.3423], and no Day x Sex interaction [F (4, 40) = 1.355; *P* = 0.2668]. Lastly, the segregated data were submitted to *post hoc* power analysis and results are shown in Supplementary Table S1. In addition, the videos for these experiments were scored by both observers, and results were compared to assess inter-rater reliability (Figure 1D). Results from the two observers were significantly correlated (Pearson's *r* = 0.9916; *P* < 0.0001). Later checks on inter-rater reliability yielded similarly high Pearson's *r* values and significant *P*-values (data not shown).

**Figure 2:**
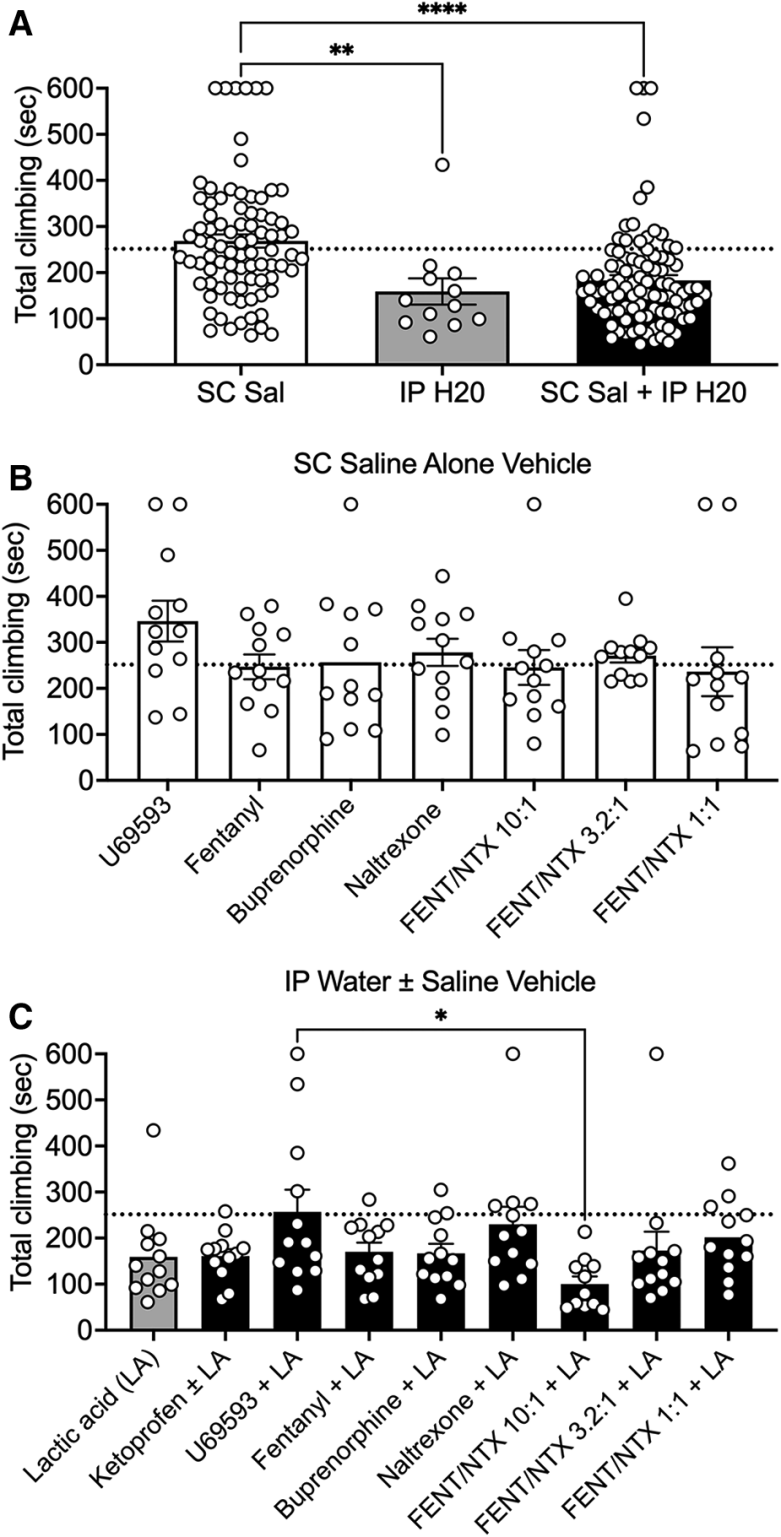
Effects of vehicle control conditions on climbing. (**A**) Comparison of climbing times for the three different types of vehicle condition. Abscissa: Type of vehicle treatment: SC Sal; *N* = 84, IP H20; *N* = 12, and SC Sal + IP H2O; N = 95. (**B**) Comparison of climbing times for each group that received SC saline as the vehicle condition. Each group is identified by the drug or drug mixture tested in the group. (**C**) Comparison of climbing times for each group that received IP water alone or SC saline + IP water as the vehicle condition. Each group is identified by the drug or drug mixture tested as a pretreatment to IP lactic acid (LA) in the group. For all panels, the ordinate is total climbing time in sec, the dotted line shows the mean climbing time on day 1 by the “No Treatment” group shown in Figure 1, bars show mean ± SEM, and points show data for individual mice. Asterisks show a significant difference between groups as indicated by one-way ANOVA and Holm-Sidak *post hoc* test. *P < 0.05, **P < 0.01, ****P < 0.0001.

Subsequent studies were conducted in 16 different groups of mice. Each of these groups included one of three types of vehicle control: (1) IP water administered alone 10 min before the session (control for IP acid alone); (2) SC saline administered alone 20 min before the session (control for studies of drugs tested alone); or (3) both SC saline and IP water (control for drugs tested as pretreatments to IP acid). Table 1 shows the mean ± SEM climbing time for the vehicle control in each group, along with the number of mice in each group that failed to meet inclusion criteria during vehicle control testing. Figure 2A compares climbing times across different vehicle controls. Climbing after SC saline alone was similar to climbing on Day 1 of the No Treatment group shown in Figure 1; however, IP water administered either alone or in conjunction with SC saline resulted in a significant decrease in climbing relative to SC saline alone [F (2, 188) = 13.08; *P* < 0.0001]. Moreover, as shown in Table 1, only 3 mice climbed less than 60 s after SC saline alone and thereby failed to meet inclusion criteria (3.4% of all mice tested), whereas 16 mice failed to meet inclusion criteria after IP water administered either alone or after SC saline (13% of all mice tested), and an additional 8 mice in these groups were excluded because IP 0.32% acid failed to produce a further decrease in climbing relative to the IP water control. To assess the stability of climbing within a vehicle control condition, Figure 2B compares climbing in the seven different groups that received SC saline alone as their vehicle control, and there was no difference in climbing across these groups [F (6, 77) = 0.9801; *P* = 0.4446]. Similarly, Figure 2C compares climbing in the nine different groups that received IP water administered either alone or in conjunction with SC saline. Although there was a significant effect of group [F (8, 98) = 2.176; *P* = 0.0357], *post hoc* analysis indicated that the only difference was between the U69593 + LA group and the FNT/NTX 10:1 + LA group. Overall, then, baseline climbing was relatively stable between groups within a given vehicle-control condition, but relative to SC saline, IP water injections resulted in higher rates of exclusion due to low climbing times, reduced climbing time in mice that met inclusion criteria, and modest but significant variation between groups.

**Figure 3:**
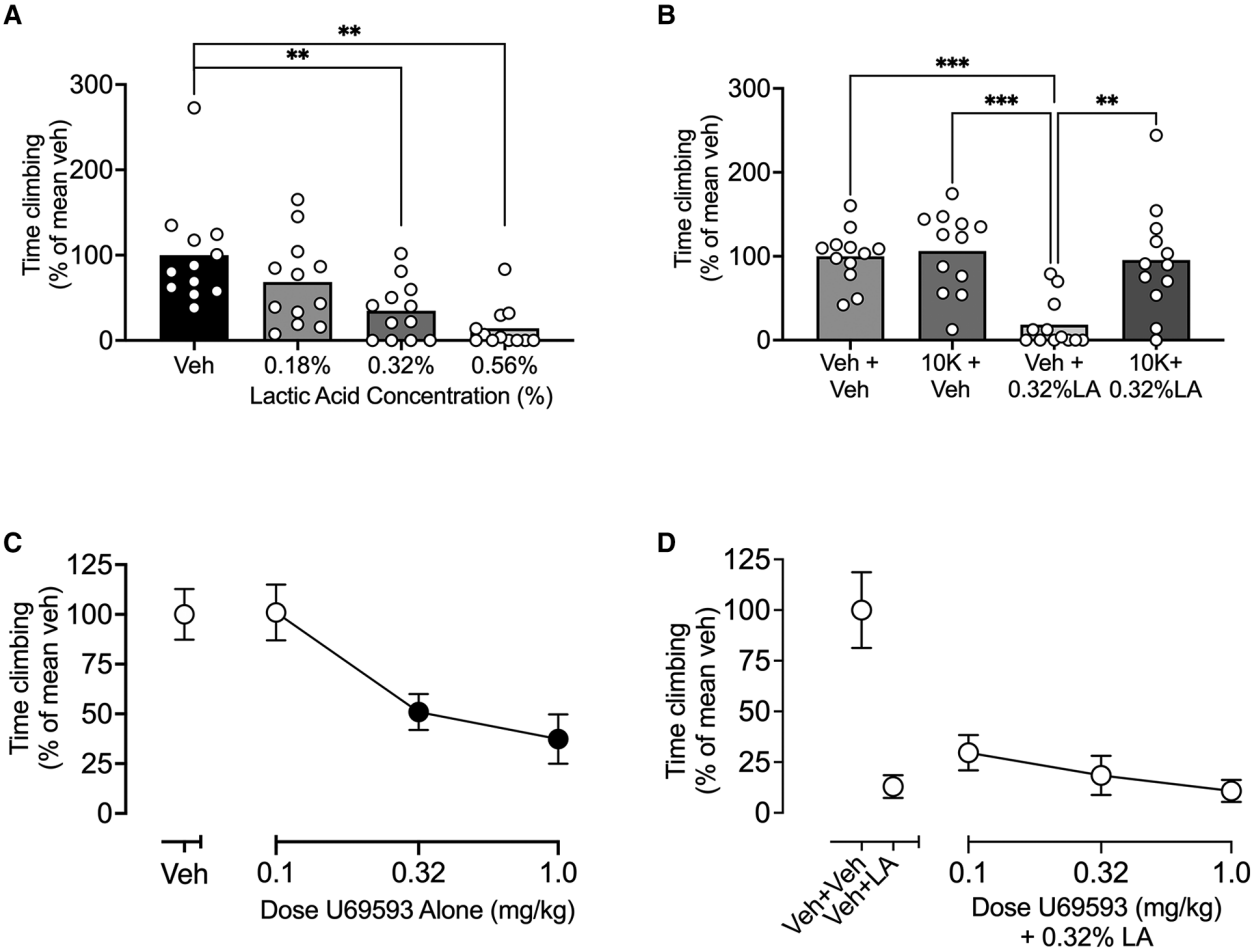
Effects of IP lactic acid, the positive control ketoprofen ± IP acid, and the negative control U69593 ± IP acid on climbing. (**A**) IP lactic acid concentration-effect curve. Abscissa: Concentration of lactic acid diluted in sterile water for IP injection. (**B**) Effects of ketoprofen ± IP acid. Abscissa: Treatment with SC saline or 10 mg/kg ketoprofen ± IP water or 0.32% lactic acid. (**C**) Effects of the kappa opioid receptor agonist U69593 administered alone. Abscissa: U69593 dose in mg/kg. (**D**) Effects U69593 administered as a pretreatment to IP 0.32% lactic acid. Abscissa: Dose of the kappa opioid receptor agonist U69593 in mg/kg. For all panels, the ordinate is time climbing expressed as percentage of the mean climbing time after vehicle (Veh in A,C; Veh + Veh in B,D) in that group, and all bars and points show mean ± SEM from 12 mice. Asterisks in panels A and B show a significant difference between treatments. **P < 0.01, ***P < 0.001. Filled points in panel C indicate a significant difference from “Veh”, *P* < 0.05. U69593 effects in panel D were compared to Veh + LA by one-way ANOVA (Veh + Veh data not included in analysis), and results were not significant.

Figure 3 shows that IP acid produced a concentration-dependent depression of climbing that could be blocked by pretreatment with ketoprofen (a positive control analgesic) but not by U69593 (a negative control non-analgesic). Thus, Figure 3A shows that IP acid produced a significant decrease in climbing at concentrations of 0.32% and 0.56% [F (2.301, 25.31) = 12.50; < 0.0001], and the concentration of 0.32% was used for all subsequent studies. Figure 3B shows that the nonsteroidal anti-inflammatory drug ketoprofen (10 mg/kg) administered alone had no effect on climbing, but it blocked IP acid-induced depression of climbing [F (2.505, 27.55) = 15.38; *P* < 0.0001]. Conversely, the kappa opioid receptor agonist U69593 (0.1–1.0 mg/kg) produced a dose-dependent decrease in climbing when it was administered alone {Figure 3C, [F (2.853, 65.62) = 21.29; *P* < 0.0001]} and failed to block IP acid-induced depression of climbing {Figure 3D, [F (2.575, 28.33) = 1.130; *P* = 0.3479]}. Statistical analysis of all Figure 3 experiments segregated by sex is shown in Supplementary Table S2. There were no sex x dose interactions for any experiment; however, there was a main effect of sex in the U69593 + LA group [F (1, 10) = 12.90; *P* = 0.0049], with males climbing less than females.

**Figure 4:**
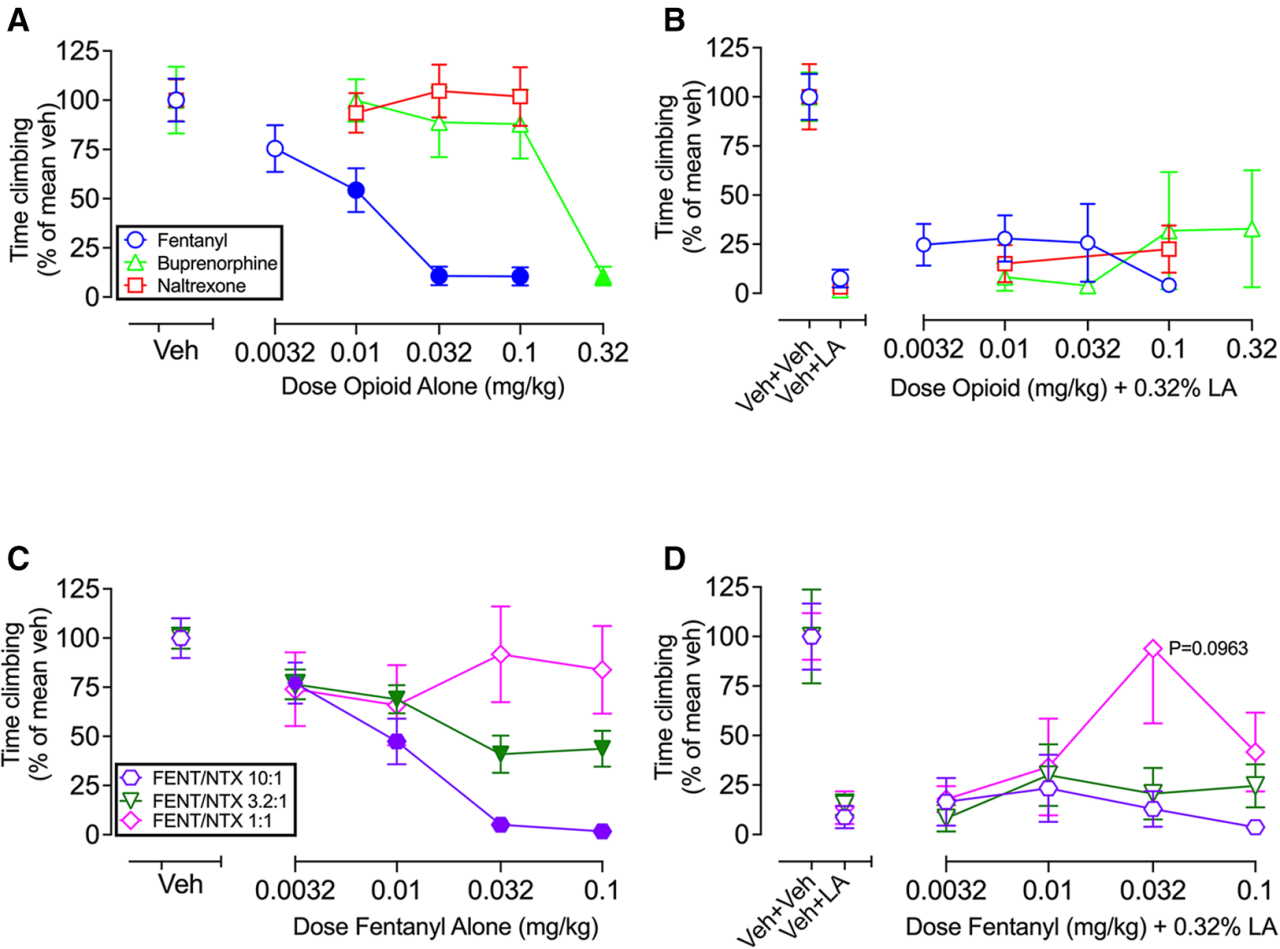
Effects of single-molecule opioids ± IP acid, and fentanyl/naltrexone (FENT/NTX) mixtures ± IP acid. (**A**) Effects of fentanyl, buprenorphine, and naltrexone administered alone. Abscissa: Dose of opioid alone in mg/kg. (**B**) Effects of fentanyl, buprenorphine, and naltrexone as pretreatments to IP 0.32% lactic acid. Abscissa: Dose of opioid in mg/kg. (**C**) Effects of 10:1, 3.2:1, and 1:1 FENT/NTX mixtures administered alone. Abscissa: Dose of fentanyl alone in mg/kg, with naltrexone dose varying according to the designated proportion. (**D**) Effects of 10:1, 3.2:1, and 1:1 FENT/NTX mixtures as pretreatments to IP 0.32% lactic acid. Abscissa: Dose of fentanyl in mg/kg, with naltrexone dose varying according to the designated proportion. For all panels, the ordinate is time climbing expressed as percentage of the mean climbing time after vehicle (Veh in A,C; Veh + Veh in B,D) in that group, and all bars and points show mean ± SEM from 12 mice, except for FENT/NTX 10:1 + IP lactic acid (*N* = 11). Filled points in panels A and C indicate a significant difference from “Veh”, *P* < 0.05. Drug effects in Panels B and D were compared to Veh + LA by one-way ANOVA (Veh + Veh data not included in analysis), and no drug effects were significant. Panel D shows that the one-way ANOVA for the 1:1 mixture approached the criterion for significance (*P* = 0.0963), and a Dunnett's *post hoc* test indicated a *P*-value of 0.1724 in comparing Veh + LA with the 0.032 mg/kg Fentanyl/0.032 mg/kg Naltrexone dose.

Figure 4 shows that single-molecule opioids (fentanyl, buprenorphine, naltrexone) and a graded series of fixed-proportion fentanyl/naltrexone mixtures (10:1, 3.2:1, 1:1 FENT/NTX) produced a dose- and efficacy-dependent decrease in climbing when they were administered alone but were ineffective to block IP acid-induced depression of climbing. Thus, Figure 4A shows dose-dependent decreases in climbing by fentanyl [F (2.634, 28.98) = 26.27; *P* < 0.0001] and buprenorphine [F (2.063, 22.70) = 10.55; *P* = 0.0005] but not by naltrexone [F (2.160, 23.76) = 0.5958; *P* = 0.5715], and Figure 4B shows that IP acid-induced depression of climbing was not alleviated by fentanyl [F (2.075, 22.83) = 0.9139; *P* = 0.4184], buprenorphine [F (1.083, 11.91) = 0.8709; *P* = 0.3781], or naltrexone [F (1.614, 17.76) = 1.113; *P* = 0.3381]. Similarly, Figure 4C shows dose-dependent decreases in climbing with fentanyl/naltrexone mixtures of 10:1 FENT/NTX [F (2.170, 23.87) = 28.79; *P* < 0.0001] and 3.2:1 FENT/NTX [F (2.794, 30.74) = 19.95; *P* < 0.0001] but not by 1:1 FENT/NTX [F (2.592, 28.52) = 1.157; *P* = 0.3388], and Figure 4D shows that IP acid-induced depression of climbing was not significantly alleviated by the 10:1 mixture [F (1.617, 16.17) = 1.188; *P* = 0.3199], 3.2:1 mixture [F (2.144, 23.59) = 0.6966; *P* = 0.5178], or 1:1 mixture [F (1.956, 21.51) = 2.627; *P* = 0.0963]. The 1:1 FENT/NTX mixture produced relatively high climbing times in some mice at the 0.032 mg/kg fentanyl/0.032 mg/kg naltrexone dose suggestive of an antinociceptive effect, but this effect did not meet the criterion for significance. Statistical analysis of all Figure 4 experiments segregated by sex is shown in Supplementary Table S3. For most groups, there was not significant main effect of sex or sex x dose interaction. However, there was a main effect of sex effect in both the buprenorphine-alone group [F (1, 10) = 7.33; *P* = 0.0220], and naltrexone + IP acid group [F (1, 10) = 6.15; *P* = 0.0325], with males climbing less than females in both groups. In addition, there was a significant sex x dose interaction in the buprenorphine-alone group [F (4, 40) = 3.74; *P* = 0.0112], but *post hoc* testing did not indicate a significant effect of sex at any dose.

**Figure 5:**
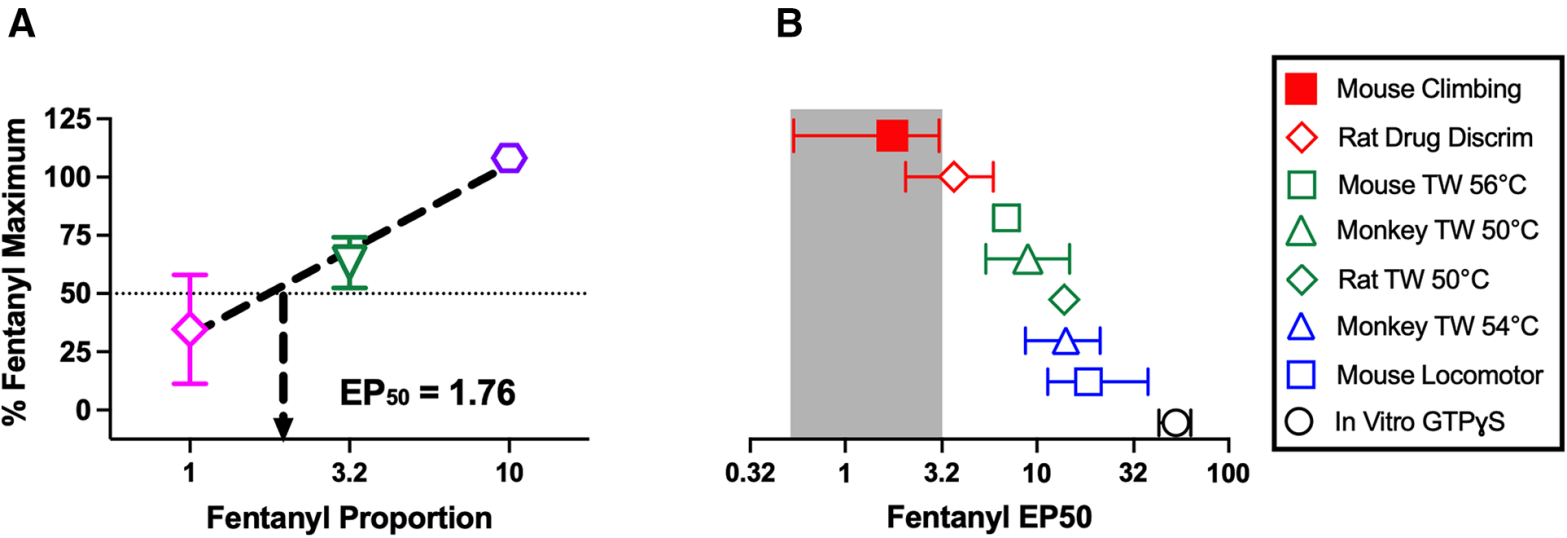
Efficacy requirement for fentanyl/naltrexone mixtures to decrease climbing. (**A**) Determination of EP50 value as a measure of opioid efficacy requirement to decrease climbing. Abscissa: Proportion of fentanyl in the mixture. Ordinate: Maximum effect of each mixture in Figure 4C expressed as a percentage of the maximum effect of fentanyl alone in Figure 4A. The efficacy requirement of the mixtures to decrease climbing can be determined by linear regression and expressed as the EP50 value, defined as the proportion of fentanyl sufficient to produce a maximum effect equal to 50% of the fentanyl-alone maximum. Points show mean ± SEM of *N* = 11–12 mice. (**B**) Comparison of EP50 values across multiple endpoints determined either *in vivo* (in the designated species) or *in vitro* (in cultured cells expressing the mouse mu opioid receptor) as reported in previous publications. Error bars show 95% confidence limits. Drug Discrim, drug discrimination; TW X°C, warm-water tail-withdrawal assay of thermal antinociception with a water temperature of X°C; GTPɣS, assay of agonist-stimulated [^35^S]GTPɣS binding.

Figure 5 shows analysis of fentanyl/naltrexone-mixture data to indicate that MOR agonist-induced disruption of climbing has a very low efficacy requirement (i.e., climbing is highly sensitive to disruption by MOR agonists administered alone). Figure 5A shows the linear regression of FENT/NTX mixture data used to determine an EP50 value as a measure of opioid efficacy to decrease climbing. Figure 5B shows that the EP50 value for decreases in climbing is lower than EP50 values for a range of other previously published *in vivo* and *in vitro* effects produced by fentanyl/naltrexone mixtures.

## Discussion

This study developed a novel assay of climbing behavior in mice and evaluated the utility of climbing as a behavioral endpoint for preclinical research on drug effects in the absence or presence of acute pain. There were three main findings. First, under baseline conditions, mice engaged in high levels of climbing that were relatively stable both across repeated testing within a group of mice and between different groups of mice. Second, climbing was depressed by IP injection of dilute acid as a visceral noxious stimulus, and this IP acid-induced depression of climbing could be blocked by the NSAID analgesic positive control ketoprofen but not by the KOR agonist negative control U69593. These findings suggest that climbing may be specifically useful as one endpoint for studies to examine effectiveness of candidate analgesics to alleviate pain-related behavioral depression. Lastly, climbing was dose-dependently reduced by MOR agonists, and analysis of results with fentanyl/naltrexone mixtures indicated that climbing in mice is more sensitive than many other behavioral endpoints to disruption by MOR agonists. These findings suggest that climbing may be especially useful for sensitive detection of undesirable motor effects of MOR agonists; however, this high sensitivity to direct effects of MOR agonists also appeared to prevent expression of an analgesic effect. Thus, climbing as assessed here illustrates the limits of MOR agonist effectiveness to restore pain-depressed behavior, and this procedure may not be useful to evaluate novel MOR agonists as candidate analgesics.

### Assessment of climbing behavior in mice

Climbing is an ethologically important component of locomotor activity for mice living in the wild, but it is rarely studied in the laboratory. Here, we assessed climbing in vertically oriented cylinders lined with wire mesh on the walls and lid, and under baseline conditions, mice engaged in climbing for ∼40% of the 10-min behavioral sessions. A few previous studies have also used vertically oriented compartments with scalable walls to assess climbing (15, 44–47). For example, one series of studies used a vertically oriented cylinder lined with horizontal bars similar to our apparatus, and in agreement with our study, climbing scores under baseline conditions were approximately 40% of the maximum possible score (15). Our study built on these earlier studies in four ways. First, we measured time climbing as a continuous ratio variable rather than assigning ordinal scores for intermittently observed climbing behavior. This increased quantitative precision, justified the use of parametric statistics for analysis, and avoided conflation of rearing and climbing behaviors. Second, our study videotaped test sessions for later scoring to avoid having an investigator in the room as an extraneous variable (48). The use of videotapes also facilitated parallel scoring by multiple observers to enable demonstration of high inter-rater reliability scores. Third, our study established stability of climbing both within individual mice during repeated testing and between different groups of mice tested over a period of months. The stability of climbing across days with individual mice justified subsequent within-subject experimental designs. The stability of climbing across multiple groups of mice treated 20-min before testing with SC saline (as a control for drug-alone studies) increased confidence that changes in climbing reflected drug effects rather than other extraneous factors that might vary across cohorts and time (note that decreases in climbing produced by 10-min pretreatment with IP water are discussed below). Lastly, our study included both male and female mice to assess the influence of sex as a biological variable (42), and sex differences were small or absent throughout the study. The role of sex as a biological variable is discussed further below.

### Climbing as an endpoint for studies of pain-depressed behavior

Results of the present study agree with previous findings that IP injection of dilute acid can serve as an acute visceral noxious stimulus to produce a concentration-dependent decrease in a range of mouse and/or rat behaviors that include feeding (20, 49), horizontal locomotor activity (9), wheel running (50), nesting (12, 18), and positively reinforced operant behavior (19, 51, 52). In the present study, 10-min pretreatment with IP water (as the vehicle control for IP acid) significantly decreased climbing relative to 20-min pretreatment with SC saline alone. In addition, a follow-up pilot study found that 10-min pretreatment with IP saline did not decrease climbing (data not shown). These findings suggest that the hypotonic water solution was sufficient to produce some behavioral disruption; nonetheless, IP acid was still effective to produce a further concentration-dependent decrease in climbing. Taken together, these instances of IP acid-induced behavioral depression can be interpreted as evidence of “pain” because (a) acid injection can produce the subjective state of pain in humans (53), (b) acid injection in humans or laboratory animals can model tissue acidosis associated with many injury- and inflammation-associated pain states (17), and (C) IP acid effects in laboratory animal studies cited above and in the present study were blocked by a clinically effective NSAID analgesic such as ketoprofen but not by a clinically ineffective negative control. To our knowledge, this is the first study to use a vertically oriented test environment with scalable walls to assess pain-related depression of climbing in mice; however, in agreement with the present results, a wide range of experimental pain models has been found to depress a potentially related behavior called “cage-lid hanging” in mice (54–56). Cage-lid hanging is assessed in horizontally oriented home-cage environments with flat floors, unscalable plastic walls, and a wire lid (54, 57), and hanging behavior occurs when mice rear or jump to the wire lid and hang from it. Moreover, as in the present study, pain-related depression of cage-lid hanging was blocked by ketoprofen but not by a centrally acting kappa opioid receptor agonist as a negative control. Overall, these results support the use of mouse climbing behavior as an endpoint for studies of pain-related behavioral depression and its pharmacological modulation by candidate analgesics.

### Effects of MOR agonists alone on climbing behavior in mice

This study showed that single molecule opioids and fentanyl/naltrexone mixtures administered alone decreased climbing behavior in an efficacy- and dose-dependent manner in ICR mice. This agrees with previous work (15), which showed that opioids potently decreased climbing behavior in an apparatus similar to the one used here. The present study builds on these previous findings by demonstrating that climbing is highly sensitive to disruption by MOR agonists and has a very low MOR efficacy requirement. Specifically, previous work in our lab has used fixed-proportion fentanyl/naltrexone mixtures as a strategy to quantify the efficacy requirements for a wide range of MOR agonist-induced behavioral endpoints in mice, rats, and rhesus monkeys (36, 38–40). Application of this approach in the present study revealed that climbing in mice is the most sensitive behavioral effect we have evaluated in any species. For example, 10-fold lower proportions of fentanyl in the fentanyl/naltrexone mixtures are sufficient to decrease climbing than to stimulate horizonal locomotion in mice, indicating that very low levels of MOR stimulation are necessary to depress climbing behavior. Climbing by mice can also be altered by some other classes of drugs, such as dopamine receptor agonists and antagonists (44, 58–60), but the relative sensitivity of climbing as a behavioral endpoint for drugs from other pharmacological classes has not been extensively evaluated. One implication of the present results is that depression of climbing is an especially sensitive endpoint for detection of behavioral impairment produced by MOR ligands, and this endpoint could be useful in characterizing the overall safety profile of MOR ligands or other drugs.

### Effects of MOR agonists as pretreatments to IP acid

MOR agonists are widely used clinically as analgesics, but in contrast to the clinically effective NSAID analgesic ketoprofen, none of the single-molecule MOR agonists or fentanyl/naltrexone mixtures was effective to alleviate IP acid-induced depression of climbing. This finding likely reflects the high sensitivity of climbing to disruption by administration of the opioids alone. In any assay of pain-depressed behavior, drug effectiveness to alleviate pain-related behavioral depression will depend on an integration of at least two effects: (1) analgesic drug effects that reduce sensitivity to the noxious stimulus and will thereby tend to increase expression of the depressed behavior, and (2) direct effects of the drug on motor function that may impair behavior and tend to exacerbate behavioral depression and obscure analgesic effects (19). In the case of the NSAID ketoprofen, there was no effect on climbing when ketoprofen was administered alone, and this enabled unobstructed expression of analgesic blockade of the IP acid-induced depression of climbing. The MOR agonists and fentanyl/naltrexone mixtures, by contrast, were both potent and effective to disrupt climbing when administered alone. As result, any blockade of IP acid effects produced by analgesic doses of these opioids was likely obscured by their direct disruption of climbing, and lower doses that did not disrupt climbing were also not sufficient to block IP acid effects.

Taken together, these results suggest that MOR agonist effectiveness to alleviate pain-related behavioral depression depends in part on sensitivity of the target behavior to disruption by the MOR agonist administered alone. Consistent with this interpretation, IP acid in mice produces a pain-related depression of both horizontal locomotor activity (9) and vertical climbing behavior (present study); however, as noted above, MOR agonists are less effective to disrupt horizontal activity than climbing and correspondingly more effective to alleviate IP acid-induced depression of horizontal activity than climbing (9). As another example, IP acid also produces a pain-related depression of positively reinforced operant behavior maintained in rats by delivery of either food or a social reinforcer (brief access to another rat); however, MOR agonists are less effective to disrupt responding maintained by food than by the social reinforcer and correspondingly more effective to alleviate IP acid-induced depression of food- than social-maintained responding (19). This interpretation has implications not only for MOR agonist effects in preclinical assays of pain-depressed behavior, but also for clinical effects of MOR agonists in humans pain patients. Pain states can interfere with a variety of behaviors in humans, and opioid analgesic effectiveness to alleviate pain-related behavioral depression may also be influenced by the behavioral endpoint of interest and the sensitivity of that endpoint to disruption by the opioid.

### Sex as a determinant of treatment effects on climbing

The present study was not intended *a priori* to detect sex differences in either basal climbing or treatment effects on climbing; however, in accordance with National Institutes of Health guidelines (61), the study did include both male and female subjects and included both inferential statistical analysis and post-hoc power analysis as we have described previously to assess the role of sex as a biological variable (42). In most test groups, there was not a main effect of sex or a significant sex x dose interaction, which implies little or no role of sex as determinant of climbing. However, this conclusion should be considered tentative because *post hoc* power analysis indicated that power was often less than the criterion level of 0.8 commonly required to protect against a Type II error (i.e., concluding the absence of a sex difference when one is present). In the three groups that did display a main effect of sex, the males climbed less than the females. More generally, the power analyses can be used to guide future studies that do choose to investigate sex differences in mouse climbing behavior.

## Data Availability

The original contributions presented in the study are included in the article/**Supplementary Material**, further inquiries can be directed to the corresponding author.

## References

[1] Cleeland CS Ryan KM. Pain assessment: global use of the brief pain inventory. Ann Acad Med Singap. (1994) 23(2):129–38. PMID: . 8080219

[2] Dworkin RH Turk DC Farrar JT Haythornthwaite JA Jensen MP Katz NP Core outcome measures for chronic pain clinical trials: IMMPACT recommendations. Pain. (2005) 113(1):9–19. 10.1016/j.pain.2004.09.012 15621359

[3] Negus SS Bilsky EJ Do Carmo GP Stevenson GW. Rationale and methods for assessment of pain-depressed behavior in preclinical assays of pain and analgesia. Methods Mol Biol. (2010) 617:79–91. 10.1007/978-1-60327-323-7_7 20336415 PMC5788447

[4] Negus SS. Core outcome measures in preclinical assessment of candidate analgesics. Pharmacol Rev. (2019) 71(2):225–66. 10.1124/pr.118.017210 30898855 PMC6448246

[5] Yu D. Translational research: current status, challenges and future strategies. Am J Transl Res. (2011) 3(5):422–33. PMID: . 22046484 PMC3204888

[6] González-Cano R Montilla-García Á Ruiz-Cantero MC Bravo-Caparrós I Tejada MÁ Nieto FR The search for translational pain outcomes to refine analgesic development: where did we come from and where are we going? Neurosci Biobehav Rev. (2020) 113:238–61. 10.1016/j.neubiorev.2020.03.004 32147529

[7] Cobos EJ Ghasemlou N Araldi D Segal D Duong K Woolf CJ. Inflammation-induced decrease in voluntary wheel running in mice: a non-reflexive test for evaluating inflammatory pain and analgesia. Pain. (2012) 153(4):876–84. 10.1016/j.pain.2012.01.016 22341563 PMC3319437

[8] Negus SS. Expression and treatment of pain-related behavioral depression. Lab Anim. (2013) 42(8):292–300. 10.1038/laban.255 PMC5425249 23877610

[9] Stevenson GW Cormier J Mercer H Adams C Dunbar C Negus SS Targeting pain-depressed behaviors in preclinical assays of pain and analgesia: drug effects on acetic acid-depressed locomotor activity in ICR mice. Life Sci. (2009) 85(7–8):309–15. 10.1016/j.lfs.2009.06.006 19559034 PMC2761814

[10] Hasriadi Dasuni Wasana PW Vajragupta O Rojsitthisak P Towiwat P. Automated home-cage monitoring as a potential measure of sickness behaviors and pain-like behaviors in LPS-treated mice. PLoS One. (2021) 16(8):e0256706. 10.1371/journal.pone.0256706 34449819 PMC8396795

[11] Jirkof P. Burrowing and nest building behavior as indicators of well-being in mice. J Neurosci Methods. (2014) 234:139–46. 10.1016/j.jneumeth.2014.02.001 24525328

[12] Diester CM Santos EJ Moerke MJ Negus SS. Behavioral battery for testing candidate analgesics in mice. I. validation with positive and negative controls. J Pharmacol Exp Ther. (2021) 377(2):232–41. doi: 10.1124/jpet.120.000464 10.1124/jpet.120.000464 PMC8058504 33622770

[13] Makowska IJ Weary DM. The importance of burrowing, climbing and standing upright for laboratory rats. R Soc Open sci. (2016) 3(6):160136. 10.1098/rsos.160136 27429772 PMC4929907

[14] Innes J Kelly C Fitzgerald N Warnock M Waas J. Detection of wild house mice and other small mammals up trees and on the ground in New Zealand native forest. N Z J Zool. (2018) 45(3):227–37. 10.1080/03014223.2018.1461660

[15] Marcais-Collado H Chaillet P Costentin J. Inhibition of the spontaneous climbing behavior elicited in mice by opiates. J Pharmacol Exp Ther. (1983) 227(2):466–71. PMID: . 6313904

[16] Urban R Scherrer G Goulding EH Tecott LH Basbaum AI. Behavioral indices of ongoing pain are largely unchanged in male mice with tissue or nerve injury-induced mechanical hypersensitivity. Pain. (2011) 152(5):990–1000. 10.1016/j.pain.2010.12.003 21256675 PMC3079194

[17] Reeh PW Steen KH. Chapter 8. Tissue acidosis in nociception and pain. In: Kumazawa T, Kruger L, Mizumura K, editors. The polymodal receptor: a gateway to pathological pain. New York, NY: Elsevier (1996). p. 143–141.

[18] Negus SS Neddenriep B Altarifi AA Carroll FI Leitl MD Miller LL. Effects of ketoprofen, morphine, and kappa opioids on pain-related depression of nesting in mice. Pain. (2015) 156(6):1153–60. doi: 10.1097/j.pain.0000000000000171 10.1097/j.pain.0000000000000171 PMC4766843 25827812

[19] Baldwin AN Banks ML Marsh SA Townsend EA Venniro M Shaham Y Acute pain-related depression of operant responding maintained by social interaction or food in male and female rats. Psychopharmacology. (2022) 239(2):561–72. 10.1007/s00213-021-06048-7 35043215 PMC10053137

[20] Stevenson GW Bilsky EJ Negus SS. Targeting pain-suppressed behaviors in preclinical assays of pain and analgesia: effects of morphine on acetic acid-suppressed feeding in C57BL/6J mice. J Pain. (2006) 7(6):408–16. 10.1016/j.jpain.2006.01.447 16750797

[21] Pande AC Pyke RE Greiner M Cooper SA Benjamin R Pierce MW. Analgesic efficacy of the κ-receptor agonist, enadoline, in dental surgery pain. Clin Neuropharmacol. (1996) 19(1):92–7. 10.1097/00002826-199619010-00009 8867523

[22] Wilkerson JL Curry ZA Kinlow PD Mason BL Hsu KL van der Stelt M Evaluation of different drug classes on transient sciatic nerve injury–depressed marble burying in mice. Pain. (2018) 159(6):1155–65. 10.1097/j.pain.0000000000001199 29528965 PMC5955845

[23] Negus SS Morrissey EM Rosenberg M Cheng K Rice KC. Effects of kappa opioids in an assay of pain-depressed intracranial self-stimulation in rats. Psychopharmacology. (2010) 210(2):149–59. 10.1007/s00213-009-1770-6 20101391 PMC3156454

[24] Lazenka MF. Antinociceptive effects of kappa-opioid receptor agonists. In: Liu-Chen LY, Inan S, editors. The Kappa Opioid Receptor.Handbook of experimental pharmacology. Cham: Springer International Publishing (2021)149–59. doi: 10.1007/978-3-030-89074-2

[25] Prescription Opioids | Drug Overdose | CDC Injury Center [Internet]. 2019 [cited 2020 Jul 6]. Available from: https://www.cdc.gov/opioids/basics/prescribed.html

[26] Hong D Flood P Diaz G. The side effects of morphine and hydromorphone patient-controlled analgesia. Anesth Analg. (2008) 107(4):1384–9. 10.1213/ane.0b013e3181823efb 18806056

[27] Nafziger AN Barkin RL. Opioid therapy in acute and chronic pain. J Clin Pharmacol. (2018) 58(9):1111–22. 10.1002/jcph.1276 29985526

[28] Ehrlich AT Darcq E. Recommending buprenorphine for pain management. Pain Manag. (2019) 9(1):13–6. 10.2217/pmt-2018-0069 30507294

[29] White LD Hodge A Vlok R Hurtado G Eastern K Melhuish TM. Efficacy and adverse effects of buprenorphine in acute pain management: systematic review and meta-analysis of randomised controlled trials. Br J Anaesth. (2018) 120(4):668–78. 10.1016/j.bja.2017.11.086 29576108

[30] Davis MP. Twelve reasons for considering buprenorphine as a frontline analgesic in the management of pain. J Support Oncol. (2012) 10(6):209–19. 10.1016/j.suponc.2012.05.002 22809652

[31] Dowell D Ragan K Jones C Badlwin G Chou R. CDC cclinical practice guideline for prescribing opioids for pain — United States, 2022. MMWR Recomm Rep. (2022) 71:1–95. doi: 10.15585/mmwr.rr7103a1 36327391 10.15585/mmwr.rr7103a1 PMC9639433

[32] Altarifi AA Yuan Y Zhang Y Selley DE Negus SS. Effects of the novel, selective and low-efficacy mu opioid receptor ligand NAQ on intracranial self-stimulation in rats. Psychopharmacology. (2015) 232(4):815–24. 10.1007/s00213-014-3719-7 25178814 PMC4310756

[33] Chakraborty S DiBerto JF Faouzi A Bernhard SM Gutridge AM Ramsey S A novel mitragynine analog with low-efficacy mu opioid receptor agonism displays antinociception with attenuated adverse effects. J Med Chem. (2021) 64(18):13873–92. 10.1021/acs.jmedchem.1c01273 34505767 PMC8530377

[34] Altarifi AA Rice KC Negus SS. Effects of μ-opioid receptor agonists in assays of acute pain-stimulated and pain-depressed behavior in male rats: role of μ-agonist efficacy and noxious stimulus intensity. J Pharmacol Exp Ther. (2015) 352(2):208–17. 10.1124/jpet.114.219873 25406170 PMC4293439

[35] Garner JB Marshall LS Boyer NM Alapatt V Miller LL. Effects of ketoprofen and morphine on pain-related depression of nestlet shredding in male and female mice. Front Pain Res. (2021) 2:673940. 10.3389/fpain.2021.673940 PMC8415797 34485976

[36] Santos EJ Banks ML Negus SS. Role of efficacy as a determinant of locomotor activation by mu opioid receptor ligands in female and male mice. J Pharmacol Exp Ther. (2022) 382(1):44–53. 10.1124/jpet.121.001045 35489781 PMC9341253

[37] Selley DE Liu Q Childers SR. Signal transduction correlates of mu opioid agonist intrinsic efficacy: receptor-stimulated [35S]GTPγS binding in mMOR-CHO cells and rat thalamus. J Pharmacol Exp Ther. (1998) 285:496–505. PMID: 9580589

[38] Selley DE Banks ML Diester CM Jali AM Legakis LP Santos EJ Manipulating pharmacodynamic efficacy with agonist+antagonist mixtures: in vitro and in vivo studies with opioids and cannabinoids. J Pharmacol Exp Ther. (2021) 376(3):374–84. 10.1124/jpet.120.000349 33443077 PMC7919866

[39] Schwienteck KL Faunce KE Rice KC Obeng S Zhang Y Blough BE Effectiveness comparisons of G-protein biased and unbiased mu opioid receptor ligands in warm water tail-withdrawal and drug discrimination in male and female rats. Neuropharmacology. (2019) 150:200–9. 10.1016/j.neuropharm.2019.01.020 30660628 PMC6476319

[40] Cornelissen JC Obeng S Rice KC Zhang Y Negus SS Banks ML. Application of receptor theory to the design and use of fixed-proportion mu-opioid agonist and antagonist mixtures in rhesus monkeys. J Pharmacol Exp Ther. (2018) 365(1):37–47. 10.1124/jpet.117.246439 29330156 PMC5830633

[41] Tuttle AH Philip VM Chesler EJ Mogil JS. Comparing phenotypic variation between inbred and outbred mice. Nat Methods. (2018) 15(12):994–6. 10.1038/s41592-018-0224-7 30504873 PMC6518396

[42] Diester CM Banks ML Neigh GN Negus SS. Experimental design and analysis for consideration of sex as a biological variable. Neuropsychopharmacol. (2019) 44(13):2159–62. 10.1038/s41386-019-0458-9 PMC6897955 31277076

[43] Faul F Erdfelder E Lang AG Buchner A. G*power 3: a flexible statistical power analysis program for the social, behavioral, and biomedical sciences. Behav Res Methods. (2007) 39(2):175–91. 10.3758/BF03193146 17695343

[44] Protais P Costentin J Schwartz JC. Climbing behavior induced by apomorphine in mice: a simple test for the study of dopamine receptors in striatum. Psychopharmacology. (1976) 50(1):1–6. 10.1007/BF00634146 827755

[45] Deacon RMJ Rawlins JNP. Hippocampal lesions, species-typical behaviours and anxiety in mice. Behav Brain Res. (2005) 156(2):241–9. 10.1016/j.bbr.2004.05.027 15582110

[46] Layne JN. Climbing behavior of peromyscus floridanus and peromyscus gossypinus. J Mammal. (1970) 51(3):580–591. 10.2307/1378397

[47] Mori T Okimoto N Sakai A Okazaki Y Nakura N Notomi T Climbing exercise increases bone mass and trabecular bone turnover through transient regulation of marrow osteogenic and osteoclastogenic potentials in mice. J Bone Miner Res. (2003) 18(11):2002–9. 10.1359/jbmr.2003.18.11.2002 14606513

[48] Sorge RE Martin LJ Isbester KA Sotocinal SG Rosen S Tuttle AH Olfactory exposure to males, including men, causes stress and related analgesia in rodents. Nat Methods. (2014) 11(6):629–32. 10.1038/nmeth.2935 24776635

[49] Kwilasz AJ Negus SS. Dissociable effects of the cannabinoid receptor agonists Δ9-tetrahydrocannabinol and CP55940 on pain-stimulated versus pain-depressed behavior in rats. J Pharmacol Exp Ther. (2012) 343(2):389–400. 10.1124/jpet.112.197780 22892341 PMC3477211

[50] Miller LL Picker MJ Schmidt KT Dykstra LA. Effects of morphine on pain-elicited and pain-suppressed behavior in CB1 knockout and wildtype mice. Psychopharmacology. (2011) 215(3):455–65. 10.1007/s00213-011-2232-5 21373789 PMC3160632

[51] Brust TF Morgenweck J Kim SA Rose JH Locke JL Schmid CL Biased agonists of the kappa opioid receptor suppress pain and itch without causing sedation or dysphoria. Sci Signal. (2016) 9(456):ra117. 10.1126/scisignal.aai8441 27899527 PMC5231411

[52] Carmo GPD Stevenson GW Carlezon WA Negus SS. Effects of pain- and analgesia-related manipulations on intracranial self-stimulation in rats: further studies on pain-depressed behavior. Pain. (2009) 144(1–2):170–7. 10.1016/j.pain.2009.04.010 19435650 PMC2717894

[53] Law LAF Sluka KA McMullen T Lee J Arendt-Nielsen L Graven-Nielsen T. Acidic buffer induced muscle pain evokes referred pain and mechanical hyperalgesia in humans. Pain. (2008) 140(2):254–64. 10.1016/j.pain.2008.08.014 18835099 PMC2613646

[54] Zhang H Lecker I Collymore C Dokova A Pham MC Rosen SF Cage-lid hanging behavior as a translationally relevant measure of pain in mice. Pain. (2021) 162:1416–25. doi: 10.1097/j.pain.0000000000002127 10.1097/j.pain.0000000000002127 PMC8054539 33230005

[55] Roemers P Hulst Y van Heijningen S van Dijk G van Heuvelen MJG De Deyn PP Inducing physical inactivity in mice: preventing climbing and reducing cage size negatively affect physical fitness and body composition. Front Behav Neurosci. (2019) 13:221. 10.3389/fnbeh.2019.00221 31680890 PMC6797814

[56] Falk S Gallego-Pedersen S Petersen NC. Grid-climbing behaviour as a pain measure for cancer-induced bone pain and neuropathic pain. *In Vivo*. (2017) 31:619–23. doi: 10.21873/invivo.11102 10.21873/invivo.11102 PMC5566911 28652428

[57] Pitzer C Kuner R Tappe-Theodor A. Voluntary and evoked behavioral correlates in neuropathic pain states under different social housing conditions. Mol Pain. (2016) 12:174480691665663. 10.1177/1744806916656635 PMC4956152 27306409

[58] Marcais H Protais P Costentin J Schwartz JC. A gradual score to evaluate the climbing behaviour elicited by apomorphine in mice. Psychopharmacology. (1978) 56(2):233–4. 10.1007/BF00431856 417378

[59] Costall B Eniojukan JF Naylor RJ. Spontaneous climbing behaviour of mice, its measurement and dopaminergic involvement. Eur J Pharmacol. (1982) 85(2):125–32. 10.1016/0014-2999(82)90457-5 7151866

[60] Kim HS Rhee GS Jung JY Lee JH Jang CG Park WK. Inhibition by noncompetitive nmda receptor antagonists of apomorphine-induced climbing behavior in mice. Life Sci. (1996) 58(17):1397–402. 10.1016/0024-3205(96)00109-9 8622565

[61] Miller LR Marks C Becker JB Hurn PD Chen W Woodruff T Considering sex as a biological variable in preclinical research. FASEB J. (2017) 31(1):29–34. 10.1096/fj.201600781r 27682203 PMC6191005

