# Supplementary material for: Climbing behavior by mice as an endpoint for preclinical assessment of drug effects in the absence and presence of pain
Source: Front Pain Res (Lausanne). 2023 Apr 17;4:1150236. doi: 10.3389/fpain.2023.1150236 (PMC10149664; doi:10.3389/fpain.2023.1150236)
Supplement: Supplementary file 1 [file Table1.docx]

Supplementary Material

**Climbing Behavior by Mice as an Endpoint for Preclinical Assessment**

**of Drug Effects in the Absence and Presence of Pain**

**Supplementary Table 1**: Two-way ANOVA results with power analysis for Figure 1.

| **Dependent measure** | **Partial eta2** | **F statistic, p value** | **Cohen’s Effect Size (Cohen’s F)** | **Current Power** | **Sample Size: Power≥0.8** |
| --- | --- | --- | --- | --- | --- |
| Day Main Effect | 0.021 | F (2.33, 23.30) = 0.22; p=0.8378 | 0.147 | 0.153 | 84 |
| Sex Main Effect | 0.124 | F (1, 10) = 0.99; p=0.3423 | 0.375 | 0.651 | 17 |
| Day x Sex Interaction | 0.119 | F (4, 40) = 1.36; p=0.2668 | 0.368 | 0.878 | 11 |

**Supplementary Table 2:** Two-way ANOVA results with power analysis for Figure 3.

| **Treatment** | **Dependent measure** | **Partial eta2** | **F statistic, p value** | **Cohen’s Effect Size (Cohen’s F)** | **Current Power** | **Sample Size: Power≥0.8** |
| --- | --- | --- | --- | --- | --- | --- |
| **Lactic acid (LA)** | Dose Main Effect | 0.578 | F (2.38, 23.75) = 13.69; P<0.0001 | 1.170 | 1 | 10 |
|  | Sex Main Effect | 0.103 | F (1, 10) = 1.04; P=0.3315 | 0.339 | 0.563 | 20 |
|  | Dose x Sex Interaction | 0.170 | F (3, 30) = 2.04; P=0.1288 | 0.452 | 0.950 | 12 |
| **Ketoprofen** | Dose Main Effect | 0.021 | F (1, 10) = 0.22; P=0.6522 | 0.147 | 0.152 | 96 |
|  | Sex Main Effect | 0.289 | F (1, 10) = 2.10; P=0.1779 | 0.639 | 0.977 | 8 |
|  | Dose x Sex Interaction | 0.101 | F (1, 10) = 1.12; P=0.3148 | 0.335 | 0.553 | 21 |
| **Ketoprofen + LA** | Dose Main Effect | 0.635 | F (1, 10) = 17.43; P=0.0019 | 1.320 | 1 | 5 |
|  | Sex Main Effect | 0.042 | F (1, 10) = 0.27; P=0.6127 | 0.209 | 0.258 | 49 |
|  | Dose x Sex Interaction | 0.062 | F (1, 10) = 0.66; P=0.4360 | 0.257 | 0.363 | 33 |
| **U69593** | Dose Main Effect | 0.558 | F (2.03, 20.27) = 12.63; P=0.0003 | 1.124 | 1 | 4 |
|  | Sex Main Effect | 0.022 | F (1, 10) = 0.35; P=0.5686 | 0.150 | 0.156 | 92 |
|  | Dose x Sex Interaction | 0.049 | F (3, 30) = 0.52; P=0.6725 | 0.228 | 0.390 | 29 |
| **U69593 + LA** | Dose Main Effect | 0.099 | F (2.45, 24.45) = 1.09; P=0.3615 | 0.331 | 0.652 | 17 |
|  | Sex Main Effect | 0.120 | F (1, 10) = 12.90; P=0.0049 | 0.370 | 0.637 | 17 |
|  | Dose x Sex Interaction | 0.060 | F (3, 30) = 0.6342; P=0.5988 | 0.251 | 0.469 | 24 |

**Supplementary Table 3:** Two-way ANOVA results with power analysis for Figure 4.

| **Treatment** | **Dependent measure** | **Partial eta2** | **F statistic, p value** | **Cohen’s Effect Size (Cohen’s F)** | **Current Power** | **Sample Size: Power≥0.8** |
| --- | --- | --- | --- | --- | --- | --- |
| **Fentanyl** | Dose Main Effect | 0.722 | F (2.68, 26.76) = 26.03; p<0.0001 | 1.613 | 1 | 3 |
|  | Sex Main Effect | 0.174 | F (1, 10) = 3.27; p=0.1006 | 0.459 | 0.818 | 12 |
|  | Dose x Sex Interaction | 0.082 | F (4, 40) = 0.90; p=0.4736 | 0.299 | 0.695 | 15 |
| **Buprenorphine** | Dose Main Effect | 0.568 | F (2.50, 25.00) = 13.17; p<0.0001 | 1.148 | 1 | 3 |
|  | Sex Main Effect | 0.351 | F (1, 10) = 7.33; p=0.0220 | 0.736 | 0.995 | 7 |
|  | Dose x Sex Interaction | 0.272 | F (4, 40) = 3.74; p=0.0112 | 0.611 | 0.999 | 5 |
| **Naltrexone** | Dose Main Effect | 0.055 | F (2.08, 20.76) = 0.58; P=0.5761 | 0.240 | 0.346 | 33 |
|  | Sex Main Effect | 0.156 | F (1, 10) = 0.39; p=0.5427 | 0.430 | 0.766 | 14 |
|  | Dose x Sex Interaction | 0.063 | F (3, 30) = 0.67; p=0.5789 | 0.258 | 0.490 | 23 |
| **FENT/NTX 10:1** | Dose Main Effect | 0.744 | F (1.95, 19.53) = 29.07; P<0.0001 | 1.705 | 1 | 4 |
|  | Sex Main Effect | 0.624 | F (1, 10) = 1.91; P=0.1970 | 1.289 | 1 | 5 |
|  | Dose x Sex Interaction | 0.099 | F (4, 40) = 1.11; P=0.3661 | 0.333 | 0.796 | 13 |
| **FENT/NTX 3.2:1** | Dose Main Effect | 0.687 | F (2.59, 25.85) = 21.97; p<0.0001 | 1.482 | 1 | 4 |
|  | Sex Main Effect | 0.118 | F (1, 10) = 0.75; p=0.4079 | 0.366 | 0.628 | 18 |
|  | Dose x Sex Interaction | 0.174 | F (4, 40) = 2.11; p=0.0971 | 0.460 | 0.980 | 8 |
| **FENT/NTX 1:1** | Dose Main Effect | 0.105 | F (2.62, 26.18) = 1.17; P=0.3357 | 0.342 | 0.677 | 16 |
|  | Sex Main Effect | 0.0002 | F (1, 10) = 0.0007; p=0.9783 | 0.015 | 0.051 | >100 |
|  | Dose x Sex Interaction | 0.102 | F (4, 40) = 1.14; p=0.3536 | 0.337 | 0.807 | 13 |
| **Fentanyl + LA** | Dose Main Effect | 0.084 | F (2.10, 20.95) = 0.91; P=0.4218 | 0.302 | 0.491 | 23 |
|  | Sex Main Effect | 0.040 | F (1, 10) = 2.04; p=0.1837 | 0.203 | 0.247 | 51 |
|  | Dose x Sex Interaction | 0.088 | F (4, 40) = 0.96; p=0.4399 | 0.310 | 0.728 | 15 |
| **Buprenorphine + LA** | Dose Main Effect | 0.080 | F (1.08, 10.78) = 0.87; P=0.3812 | 0.294 | 0.321 | 38 |
|  | Sex Main Effect | 0.073 | F (1, 10) = 1.17; P=0.3045 | 0.281 | 0.421 | 28 |
|  | Dose x Sex Interaction | 0.085 | F (4, 40) = 0.93; P=0.4591 | 0.304 | 0.710 | 15 |
| **Naltrexone + LA** | Dose Main Effect | 0.105 | F (1.37, 13.65) = 1.17; P=0.3186 | 0.342 | 0.546 | 21 |
|  | Sex Main Effect | 0.154 | F (1, 10) = 6.15; P=0.0325 | 0.426 | 0.758 | 14 |
|  | Dose x Sex Interaction | 0.135 | F (2, 20) = 1.56; P=0.2340 | 0.395 | 0.801 | 13 |
| **FENT/NTX 10:1 + LA** | Dose Main Effect | 0.129 | F (1.52, 13.63) = 1.33; P=0.2877 | 0.384 | 0.554 | 19 |
|  | Sex Main Effect | 0.197 | F (1, 9) = 1.16; P=0.3100 | 0.496 | 0.832 | 11 |
|  | Dose x Sex Interaction | 0.102 | F (4, 36) = 1.03; P=0.4065 | 0.338 | 0.763 | 13 |
| **FENT/NTX 3.2:1 + LA** | Dose Main Effect | 0.063 | F (2.01, 20.08) = 0.672; P=0.5226 | 0.259 | 0.367 | 31 |
|  | Sex Main Effect | 0.005 | F (1, 10) = 0.109; P=0.7486 | 0.074 | 0.075 | >100 |
|  | Dose x Sex Interaction | 0.057 | F (4, 40) = 0.604; P=0.6618 | 0.246 | 0.502 | 22 |
| **FENT/NTX 1:1 + LA** | Dose Main Effect | 0.208 | F (2.02, 20.19) = 2.63; P=0.0962 | 0.513 | 0.921 | 10 |
|  | Sex Main Effect | 0.045 | F (1, 10) = 0.752; P=0.4062 | 0.216 | 0.274 | 45 |
|  | Dose x Sex Interaction | 0.092 | F (4, 40) = 1.01; P=0.4131 | 0.318 | 0.753 | 14 |
|  |  |  |  |  |  |  |
